# Simple, Fast and Efficient Methods for Analysing the Structural, Ultrastructural and Cellular Components of the Cell Wall

**DOI:** 10.3390/plants11070995

**Published:** 2022-04-05

**Authors:** Renan Falcioni, Thaise Moriwaki, Renato Herrig Furlanetto, Marcos Rafael Nanni, Werner Camargos Antunes

**Affiliations:** 1 Department of Agronomy, State University of Maringá, Av. Colombo, 5790, Maringá 87020-900, PR, Brazil or pg52885@uem.br (T.M.); pg53830@uem.br (R.H.F.); mrnanni@uem.br (M.R.N.); wcantunes@uem.br (W.C.A.); 2 Department of Biology, Paraná Federal Institute of Education, Science and Technology, Avenida Bento Munhoz da Rocha, PRT 280, s/n°, Trevo Codapar, Palmas 85555-000, PR, Brazil

**Keywords:** biochemical analyses, cellulose and lignin, far-red light, microscopy analyses, primary and secondary cell wall, sample preparation

## Abstract

Plant cell walls are a fundamental component of plant biology and play an essential role in plant growth and development. The metabolic components of the cell wall can be investigated in a fast, simple, and highly efficient manner using various and distinct microscopy techniques. Here, we report implementing a flowchart to analyse tobacco plants’ structural, ultrastructural, and metabolic components supplemented with far-red light. In addition, biochemical components, such as lignin, cellulose, phenolic compounds, and reducing sugars, present in the plant cell walls were quantified using light, fluorescence, and electron microscopy. Our data were generated from samples prepared via tissue fixation, incorporation in resins, and slicing using microtomes. Moreover, we have used routine staining and contrast techniques to characterise plant cell walls. Here, we describe several protocols that use classic and modern techniques as well as qualitative and quantitative analytical methods to study cell walls, enabling the plant research community to understand and select the most suitable methods for the microscopic analysis of metabolic components. Finally, we discuss specific ideas aimed at new students of plant anatomy and microscopy. This research not only described the structural, ultrastructural, and metabolic components of the plant cell wall, but also explained the strategies for understanding cellular development.

## 1. Introduction

Plant cell walls are a fundamental component of plant biology and can strongly affect the quality of plant-based products used in industrial processes [1,2,3]. The invention of the first microscopes approximately 400 years ago by Hans and Zacharias Janssen in 1591, and their improvement by Robert Hooke and Antonie van Leeuwenhoek in 1620 and 1665, promoted a significant contribution to scientific paradigm changes and facilitated the description of the first cells [4]. Over time, this knowledge has improved and has been incorporated into present-day research. Microscopy techniques and methodologies were then developed and enhanced. Different markers and stains were, over time, combined to investigate plant biological structures.

Light and electron microscopy techniques are of fundamental importance as they help recognise the status, and dynamics of primary and secondary plant walls [1,5]. The advantages of using histochemical methods in conjunction with different microscopy instruments to quantify metabolites include a better understanding of the acclimation and adaptive responses of plants to distinct environments [6,7]. Cellular responses to climate change, improved biofuel production, and enhanced crop productivity can be measured using these techniques [6,8]. This enables the development of better strategies and promotes our understanding of growth and development dynamics, for example, the allocation of energy during the formation of different tissues, vegetable crop, pest resistance, reproductive rate, and structural components in the cell wall, as well as during biosynthesis [9,10,11,12].

In general, the efficiency of plant cell wall staining and different analytical techniques, including light, fluorescence, and electron microscopy, requires preparation and knowledge of the best tools to study cell wall architecture [1,13,14]. For example, several key staining, immunostaining, and contrast techniques require knowledge of acidophilic or basophilic cell components. Moreover, unique skills are required in processes such as solution preparation, infiltration, and fixation, manipulating blocks to obtain thin sections, staining, handling specialist equipment, and performing statistical analyses to investigate the histochemical and metabolic characteristics of plant cell walls as well as acquiring qualitative and quantitative data [3,15].

Plant biologists have become increasingly dependent on the main microscopy techniques to quantify the structural components of the cell wall [3,7,16], thereby accumulating information to further our understanding of different areas of research, e.g., the study of photodevelopment, the stimulation of the phenylpropanoid pathway to produce inhibitory molecules such as some phenolic compounds, changes in the bioaccumulation of pollutants and heavy metals in cell walls, as well as the evaluation of gene expression measurements in specific tissues and cells [3,5,17]. Among the hundreds of interactions and the biotic and abiotic changes occurring within a cell, cell wall formation under light conditions triggers a series of transcriptional changes that are related to photoreceptors (e.g., phytochromes and cryptochromes) or modified by different classes of plant hormones [9,10,18,19]. The exposure of the internode part of stems to far-red light has revealed that layers of cell walls can be regulated and adjusted to maintain plant growth and development [7,20,21].

**Figure 1:**
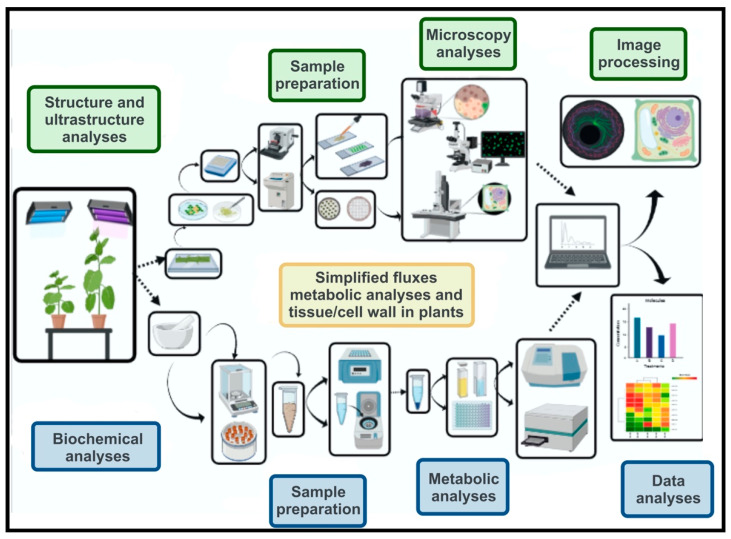
The schematic representation of an integrated analysis between the structural and ultrastructural components of the cell wall using different microscopy techniques and biochemical analyses. The collected data were processed using qualitative and quantitative analyses, following the implementation of fixation and extraction methods. The samples were processed using different tools, equipment and techniques. Structural analyses were carried out using various microscopy techniques; metabolic analyses were performed using spectrophotometers or microplate readers. The data were processed and analysed using image software and statistical packages. These procedures are described in detail in the “Materials and Methods” section.

In this communication, we present a simplified flowchart of the tools that can be used to study the cell wall, using stimulation by light or in other experiments based on the researchers’ hypotheses and creativity. Here, we describe the morphological changes that occurred in tobacco plants grown under white or white plus far-red light conditions and suggest a few quick, simple, and inexpensive methods which can be employed to acquire information during the study of important cell wall components. In addition, we also report here various analyses of the structural and ultrastructural components of the cell wall using different microscopic techniques and the quantification of biochemical components, such as lignin, cellulose, phenolic compounds, and reducing sugars, present in plant cell walls (Figure 1).

## 2. Results

### 2.1. Morphological Growth under Far-Red Light Conditions

**Figure 2:**
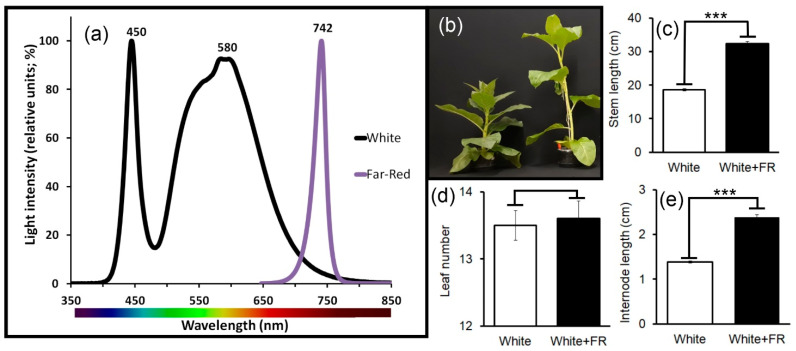
Characterisation of tobacco plants and their growth analysis under distinctive light qualities (White and White + FR) using LEDs (350 at 850 nm), 25 days after the start of treatment. (**a**) Spectral emissivity measurements of LEDs indicating peak wavelength and peak height. Data were normalised to peak = 100%. (**b**) From left to right: tobacco plants grown under White and White + FR conditions. (**c**) Stem length (cm). (**d**) Leaf number. (**e**) internode length (cm). Asterisks (***) indicate statistical differences between treatments determined by the Student’s *t*-test (*p* < 0.001); (*n* = 10 ± SE).

The variation in spectral quality, using far-red (FR) light supplementation (Figure 2a), promoted etiolation in plants (White vs. White + FR; Figure 2b). Etiolated plants produced longer stems (73.5%) and increased internode lengths (72.4%) (Figure 2c,e; *p* < 0.001). However, the total number of leaves in etiolated plants compared was not consistently higher than in plants supplemented with white light (Figure 2d; *p* > 0.001).

### 2.2. Qualitative and Quantitative Alterations in the Structural and Ultrastructural Components of the Stem Using Microscopy Analysis

**Figure 3:**
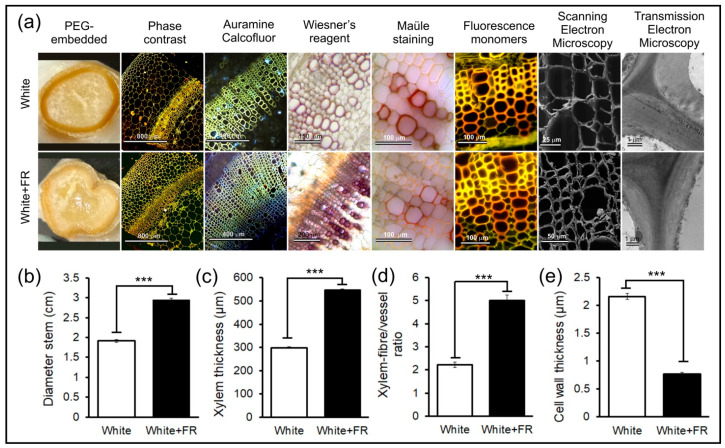
Qualitative and quantitative analyses of the structural and ultrastructural changes, as well as of the cell wall components of the stems of tobacco plants grown under White and White + FR conditions. Samples were fixed in Karnovsky’s solution, embedded in PEG 6000 and analysed using different staining techniques followed by fluorescence and electron microscopy. (**a**) From left to right: stem sections embedded in PEG 6000; stained with toluidine blue and analysed under light microscopy with phase contrast; blocks in methyl methacrylate stained with Auramine O and Calcofluor Bright White 28 and analysed using epifluorescence microscopy; lignified cells stained with Wiesner’s reagent and analysed under light microscopy; Mäule reagent showing lignin monomers under light microscopy; staining with astra blue and safranin-fuchsin followed by fluorescence microscopy indicate changes in the monomeric composition of lignin; superficial changes in the texture and structure of the cell wall revealed via scanning electron microscopy (SEM) and changes in the organisation and arrangement of cellulose microfibrils revealed via transmission electron microscopy (TEM). (**b**) Stem diameter of the medial region (cm). (**c**) Xylem thickness (µm). (**d**) Xylem fibre-like-cells/vessel ratio. (**e**) Cell wall thickness (µm). Asterisks (***) indicate statistical differences between distinct treatments using the Student’s *t*-test (*p* < 0.001); (*n* = 10 ± SE).

**Figure 4:**
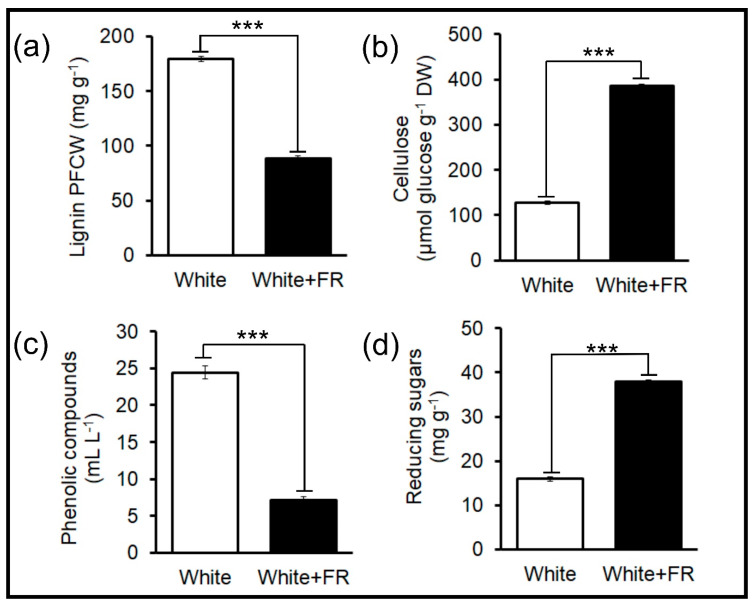
Metabolic compounds extracted from the cell wall of tobacco stems. (**a**) Lignin content in protein-free cell walls (PFCWs). (**b**) Cellulose content is equivalent to the glucose standard. (**c**) Total phenolic compounds equivalent to gallic acid. (**d**) Reducing sugars extracted from the cell wall. Asterisks (***) indicate statistical differences between treatments using the Student’s *t*-test (*p* <0.001); (*n* = 10 ± SE).

**Figure 5:**
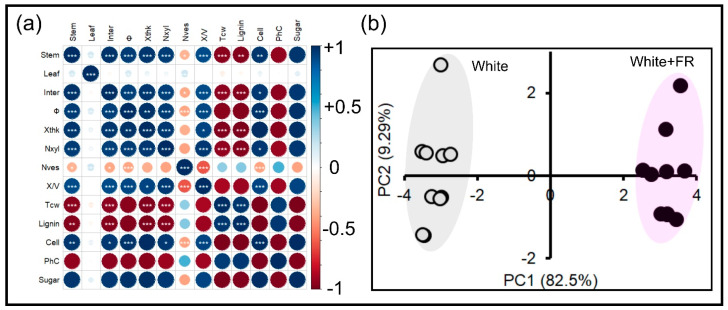
Pearson’s correlation and principal component analysis. (**a**) Measurement of morphological, structural and metabolic components using Pearson’s correlation. (**b**) Principal component analysis of samples from both White and White + FR groups. Asterisks: * *p* < 0.05, ** *p* < 0.01, *** *p* < 0.001. Measured extracts and growth variables include stem number, leaf number, internode, stem diameter (Φ), xylem thickness (Xthk), number of xylary fibres (Nxyl), number of vessels (Nves), xylary fibre/vessel ratio (X/V), thickness of cell wall (tcw), lignin concentration (Lignin), cellulose content (Cell), phenolic compounds (PhC) and reducing sugars (Sugar); (*n* = 10 ± SE). Open circle represents White and filled circle White + FR light treatment in tobacco plants.

Morphological changes in tobacco stems (at the same developmental stage) grown under White and White + FR conditions were evaluated using different methods as well as traditional microscopy techniques (Figure 2, Figure 3, Figure 4 and Figure 5).

Analysis using phase-contrast microscopy with toluidine blue showed changes in the epidermis, cortical parenchyma, vascular cylinder, and medullary parenchyma in response to photodevelopment. A greater intensity of staining of the vascular cylinder was detected in plants exposed to White light, whereas a uniform intensity was observed in samples from plants exposed to White + FR light (Figure 3a). Double staining with Auramine O and CalcoFluor Bright White 28, to detect lignin and cellulose, respectively, followed by fluorescence microscopy, revealed blue stains which indicate cellulose accumulation in primary, thin-walled parenchyma cells (cortical and medullary). Conversely, yellow staining indicated an accumulation of lignin and other phenolic compounds in thickened vessel elements, thickened xylem fibre-like cell, and the secondary cell wall of tracheids (Figure 3a).

Analysis using classic reagents, such as acidified floroglucinol (Wiesner’s reagent) or Mäule reagent, showed changes in the lignin composition in vessel elements and xylem fibres. Monomeric components such as syringyl propane (S-type, sinapyl alcohol) were stained red, whereas guaiacyl (G-type, coniferyl alcohol) and hydroxyphenyl (H-type, coumaryl alcohol) were stained brown. A red or brownish-red colouration was identified in vessel elements and a fainter yellow tone was observed in xylem fibre-like cell from plants under both White and White + FR conditions. Structural monomers of lignin present in the vessel elements, xylem fibres and tracheids were identified via the double staining of tissues using astra blue and fuchsin-basic, followed by UV fluorescence microscopy at 400 nm. This method can be used to identify differences among the H, G, and S monomers, depending on the plant’s ontogeny and the cell type being analysed (Figure 3a). Colour tones varied from red-orange for vessel elements, to yellow for xylary fibres in secondary cell walls, and/or light green in primary cell walls. In addition, other qualitative changes, as indicated by the increased cell wall thickness in the fibres and vessel elements of plants exposed to White light and by the reduced interaction and intensity of the dyes in plants exposed to White + FR light, were associated with molecular changes in the ultrastructural components and biochemical composition of the cell walls in plants (Figure 3a).

Scanning electron microscopy (SEM) and transmission electron microscopy (TEM) are useful techniques for studying the molecular, textural, and ultrastructural components of the cell wall (Figure 3a). SEM revealed that cell walls in plants exposed to White light were highly delimited and organised. However, under White + FR conditions, cell walls appeared more fragile and less organised. TEM showed that, under White conditions, cell walls were thicker, more delimited and contained cellulose microfibrils that were oriented parallel to the middle lamella. By contrast, under White + FR conditions, cell walls exhibited reduced thickness and more fragile characteristics, including cellulose microfibrils that were less organised, more spaced out, and some oriented perpendicularly in relation to the middle lamella (Figure 3a).

In addition to these qualitative observations, structural changes were quantified through measurements using free software packages such as ImageJ. Quantitative data (Figure 3b–e; *p* < 0.001) revealed that plants exposed to White + FR light exhibited increments in stem diameter and parenchymatous tissues (53.9%), xylem thickness (82.8%), and xylary fibre to vessel ratio (125.6%) that were greater compared to plants exposed to White light (Figure 3b–d). However, based on the measurements obtained for S1, S2, and S3, the cell wall thickness was reduced (64.6%) in plants grown under White + FR light conditions (Figure 3e). For example, fibres or vessel elements are made of a primary layer *p*, and secondary layers S1, S2, and S3, and each new layer deposition increases the thickness cell wall (Figure 3a).

### 2.3. Biochemical Composition of the Cell Wall

The biochemical components of the cell wall can be quickly quantified, providing accurate data on the structural and molecular development of primary and secondary walls (Figure 4 and Figure 5). The contents of lignin (Figure 4a), cellulose (Figure 4b), phenolic compounds (Figure 4c), and reducing sugars (Figure 4d) showed strong correlations (*p* < 0.001) between the White and White + FR treatments (Figure 4 and Figure 5) and were identified through principal component analysis (Figure 5).

In particular, lignin (102.8%) and phenolic compound (241.2%) levels were higher in plants under White conditions and displayed a strong and positive correlation (r = 0.954) (Figure 4a,c and Figure 5a). The highest levels of cellulose (203.1%) and reducing sugars (137.7%) were detected in plants under White + FR conditions and showed a strong correlation (r = 0.999) (Figure 4b,d and Figure 5a). Strong correlations (r > 0.89), both positive and negative, were observed between the cell wall extracts (polymeric chains of cellulose, lignin, phenolic components, and reducing sugars) and morphological (growth) variables, such as stem diameter, xylem thickness, number of xylem fibres, and cell wall thickness (Figure 5a).

The results of multivariate analysis using principal components analysis (91.8%) have distinguished the treated samples (collected from plants grown under White and White + FR conditions) were separated into two large groups, namely PC1 (82.5%) and PC2 (9.29%). These groups explained the changes among the morphological, structural, ultrastructural, and biochemical components related to the cell wall (Figure 5b).

## 3. Discussion

Tobacco plants grown under white light or white light supplemented with far-red light exhibited several morphological, structural, and ultrastructural changes, all of which were quickly determined with the use of qualitative and quantitative tools (Figure 1, Figure 2, Figure 3, Figure 4 and Figure 5). Many researchers today use microscopy techniques and choose the metabolic components of the cell wall to obtain valuable information on the dynamics of development in primary and secondary plant walls. Our data indicate that these techniques and tools can be further applied in plant research, consequently allowing resources to be directed to the development of experiments that require more expensive applications such as “omic” technologies (Figure 1).

Tobacco plants exhibit high morphological and structural phenotypic plasticity. As such, they are an excellent model plant for studying changes in the plant cell wall. In the following sections, we discuss and recommend the methods used in the microscopy analysis and quantification of cellular components. In addition, we describe the techniques and tools necessary for sample preparation and tissue sectioning using different microscopy techniques and for the quantification of lignin, cellulose, phenolic compounds, and reducing sugars in the cell wall (Figure 1, Figure 2, Figure 3, Figure 4 and Figure 5).

### 3.1. Fixative Solutions

Typically, a series of analyses can be performed using microscopic techniques to investigate the structural components and composition of the primary and secondary cell wall Figure 1 (also described in Section 4). The first and fundamental step is the rapid fixation of the target tissues, using different fixative solutions, such as formalin, ferrous sulphate-formalin, caffeine-sodium benzoate, alcohol, and acetic acid [3]. Moreover, the modified Karnovsky’s solution is neutral (pH 7–7.4), prepared in phosphate buffer and can be stored in a refrigerator. These fixatives are excellent at preserving the structural and molecular integrity of countless tissue types and molecules, without interfering with sample colour or contrast [3,9,22]. Karnovsky’s solution has proven to be one of the safest and cheapest regents, with low interference in microscopic procedures, when used together with the different techniques and methods (including analysis by light, fluorescence, and electron microscopy) to determine the anatomy of tobacco plants. Fixed samples embedded in PEG or polymeric resins produced excellent analytical results during staining and contrast procedures [3,10,23]. Therefore, in tobacco plants, these fixative methods have been shown to be highly efficient and enable to obtain higher quality images using microscopy techniques.

### 3.2. Infiltration Medium and Block Sectioning

To obtain high-quality tissue sections, and consequently excellent images, it is essential to choose the most suitable infiltration medium [5]. Consideration should be given to the type of treatment imposed and the tissue to be analysed. PEG 6000, as well as methyl methacrylate or Spurr resin, are media with excellent stability. Tissue inclusion using these media results in precise cuts on the microtome [3,15]. Plants supplemented with FR light and that exhibited significant changes in the cell wall were easily analysed when treated with these infiltration media [3]. When combining these media with different dyes, probes, markers, and contrast solutions, it is possible to analyse and identify the structural and ultrastructural changes of the primary and secondary cell wall in plants quickly, safely, and economically (Figure 3).

Tissue sections can be obtained using a semi-automatic microtome or ultramicrotome [3]. It should be noted that thinly cut fresh tissue or freehand cuts of embedded tissue are great methods for obtaining quick sections for qualitative analysis [1,3]. However, the characteristics for quantitative analysis are lost in freehand cuts, as the thickness and angle of the cut sections are not standardised. Another interesting technique is the use of a vibratome, which can be used with fresh material or material embedded in inclusion medium such as colourless gelatine [1,5]. This technique does not require extensive preparations of plant material and the angles and thickness of the cuts are predefined. However, this equipment can be relatively expensive, and several laboratories have yet to acquire it. By contrast, sections embedded with PEG 6000 or polymeric resin (methyl methacrylate or Spurr) can be cut at highly standardised thicknesses (70 nm, 1 µm, 5 µm, or 25 µm) depending on the objective of the study, are easily transferred to slides, and can be photographed under both light and fluorescence microscopes, thus requiring lower investment in equipment and reagents. Another widely used inclusion medium is paraffin. This procedure requires the tissue fragments or samples to be at higher temperatures in oven-dried or oven-vacuum, to facilitate the infiltration and fusion of the solution between the intercellular spaces of the plant material. However, although a valuable and highly recommended medium for the histological analysis of plants, paraffin can damage or deform the primary cell walls, which are thinner and more tenuous. Thus, the methods using PEG and methyl methacrylate are highly effective in tissues with both a higher or lower level of lignification, as well as in fragile tissues, when studying plant walls with thickness, lengths cells, and pattern sections in lignification stems of the tobacco plants [3,9,10].

### 3.3. Staining and Contrast Using Light and Electron Microscopy

When investigating different types of plant organs, tissues, and cells (see Section 4), the choice of dye solutions (for light and fluorescence microscopy) or contrast (for SEM and TEM) is important to analyse and identify the multiple components and molecules of the primary and secondary walls (Figure 1 and Figure 3). Tobacco, as well as other model plants, such as *Arabidopsis*, *Populus,* and *Mendicago*, produce numerous molecules that are associated with different dyes [1,3]. Care and precaution should be taken during procedures that require staining using acidic or corrosive basic solutions. These solutions should not come into contact with the microscope objective lens, as they can cause irreversible damage [24]. In addition, if they come into contact with the lens, a solution of alcohol-ketone (7:3) or xylene should be used. In contrast, several non-toxic reagents (e.g., astra-fuchsin blue, auramine and calcofluor) also exist, which can be used to analyse a wide range of cell wall components and structures (Figure 3). Plant tissues, cells, and cellular structures can be quickly identified using numerous dyes or fluorochromes. For example, the differentiation of lignin types under blue and violet lights was also observed under epifluorescence, and could furthermore be identified via fluorochromasia in safranin-stained sections [3]. Although we emphasise here the analysis of primary and secondary walls in stems using microscopy-based procedures, these techniques (see Section 4) can be similarly used to study other organs and tissues such as roots, leaves, flowers and seeds. Finally, lignin types may be successfully identified using Mäule’s test in tobacco plants for light microscopy observation or by staining with safranin-fuchsin for observation under epifluorescence microscopy, following [3,10].

In addition, the correct handling by researchers and the final disposal of waste in order not to harm the environment must also be considered [3,24]. However, many reagents that are non-toxic and allow a comprehensive response for the analysis of the composition and structures of cell walls, can be used (astra-fuchsin blue, auramine and calcofluor) (Figure 3). For example, the differentiation of lignin types under blue and violet lights was also observed under epifluorescence, and could be distinguished based on fluorochromasia in safranin-stained sections [3], following what is displayed in light treatments in tobacco plants (Figure 3). However, although we emphasise the analysis of primary and secondary walls in stems, these same techniques (see material and methods) can be used for studies of other organs and tissues, such as roots, leaves, flowers, and seeds [7]. Finally, lignin types may be successfully distinguished with Maule’s test for light microscopy observation or by staining with safranin-fuchsin for observation under epifluorescence with blue or violet excitation with fluorescence microscopy.

The microscopic analyses that require more time, experience, and costly reagents are those centred around electron microscopy (Figure 1 and Figure 3). To circumvent these limitations, university laboratories would typically own equipment such as metallised, ultramicrotomes, and scanning and transmission electron microscopes in a common space shared by users. Moreover, the reagents necessary for SEM and TEM analysis, although initially presenting high acquisition costs, have a longer shelf-life, either as stock solutions or solid reagents, when properly stored. Considering the minimal amount of reagent and solution used in the preparation of plant samples, these reagents can be used for a long time, once purchased. Among the reagents and consumables used in electron microscopy, a diamond knife is necessary for ultra-fine cuts (70 nm). Although expensive, these can be easily replaced by glass knife. However, the latter wear out more quickly and are less efficient [25].

### 3.4. Image Acquisition and Statistical Analyses

Last but not least, phase contrast, fluorescence, or scanning and transmission electron microscopy determine whether the previously mentioned procedures were suitably executed to generate high-quality images (Figure 1, Figure 2, Figure 3 and Figure 4). The images under light or fluorescence microscopy are visible via the interaction of light with matter, which shows the different colours or excitation of a given fluorochrome [1,3]. The choice of dyes allows the identification of different cellular and structural components of the cell wall, based on their affinity for basophilic or acidophilic molecules present in different tissues [24]. Conversely, electron microscopes (SEM and TEM) use electron beams to interact with samples or sections placed on grids with high contrast [10]. Images are generated due to contrasting means. In SEM, contrasts within samples occur through metallisation with gold or vaporisation with carbon. In TEM, the samples are contrasted against solutions with high molecular weights (e.g., containing lead or uranyl) [26]. These high molecular weight compounds enable the electron beam to interact with the samples in a high-vacuum chamber and generate ultrastructural images of the plant cells. Next, these images are captured with CCD-digital cameras and processed for analysis and measurement using software packages, such as ImageJ, Image-Pro-Plus^®^, or routine automated R scripts. This generates a series of data that can be analysed quantitatively [10]. Different statistical methods such as correlation matrices, cluster analysis, multiple tests and multivariate analysis (Figure 1 and Figure 5) can be used to accurately determine the physiological conditions of cell wall formation and development [27]. In addition, distinct changes promoted by light and other environmental factors can be easily analysed quantitatively and qualitatively by microscopy techniques.

### 3.5. Simplified Scheme to Determine Cell Wall and Metabolic Fluxes in Plants

Several molecules present in the cell wall can be routinely quantified using glycomics, proteomics, liquid and gas chromatography coupled with mass spectrometry, and magnetic resonance. However, before considering these more sophisticated and elaborate tools, a simpler series of analyses can be employed (Figure 1 and Figure 4). The lignocellulosic composition of cell walls can be analysed using the acetyl bromide or anthrone methods [28,29,30]. These highly efficient techniques can be used to identify the contents of components by binding to polymers within the plant walls and carry very low associated costs.

In general, primary cell walls are composed of 25% cellulose and less than 0.01% lignin or other phenolic compounds [8,31]. Secondary walls, on the other hand, may exhibit higher percentages of cellulose (50–80%) and lignin (15–35%), organised in a structure of cellulose microfibrils [7]. Because lignin is covalently linked to cellulose and other polysaccharides in the cell membrane (plasma membrane), it is essential to quantify these components (Figure 3 and Figure 4). Rapid analysis of the lignocellulosic content is necessary to recognise any ultrastructural and molecular change, the organisation and spatial disposition of cellulose microfibrils, as well as the incorporation of different monomer subunits, such as H-type, G-type, and S-type [3,10,32]. Several simple and quick methods containing one or two extraction and hydrolysis steps can be employed, in association with the classic Updegraff and acetyl bromide method [29,33]. In addition, using other variations of the classic method, more than 200 samples can be processed per week, using a microscale and a 96-/384-well plate reader [28,30,33]. These modified methods produce good yields quickly, efficiently, with low costs and high accuracy [30].

Together with cellulose, lignin is one of the most abundant polymers in the cell wall and has been extensively studied in recent years. The techniques presented here have helped characterise plant materials with varying levels of cell wall recalcitrance [17,34], which are important for biotechnological and industrial processes [35,36,37]. A greater understanding of the phenolic groups and the quantification of other reducing polysaccharides and sugars is therefore essential when performing rapid biochemical analyses in the study of plant cell dynamics.

## 4. Materials and Methods

### 4.1. Plants and Experimental Design

Tobacco (*Nicotiana tabacum* ‘Samsun NN’) plants were cultivated. Seeds were germinated on Germitest^®^ paper containing 5 mL of Hoagland’s solution (pH 5.4) in a petri dish. After 30 days, seedlings were transplanted from the commercial substrate and transferred to individual, white-covered wood boxes illuminated by LEDs with low spectral dispersion (Figure 2a). LED emissivity qualities was verified using a high-resolution spectroradiometer (FieldSpec 3; ASD Inc., Boulder, CO, USA). White light (WL) was measured at 450 nm (peak at 580 nm) and 740 nm (peak at 742 nm). Light irradiance was fixed at 200 µmol m^−2^ s^−1^, individually adjusted using a quantum sensor (LI-190R; Li-Cor Inc., Lincoln, NE, USA) under a 12-h/12-h (light/dark) photoperiod and at 25 °C. The experiment was set up (white and white + far-red light conditions, i.e., White and White + FR, respectively, at 742 nm) in a randomised factorial design. Plant growth and development were monitored throughout the assessment. On day 25, plant stem tissues were collected and freshly analysed, flash-frozen in liquid nitrogen, freezing-dry or oven-dried (at 70 °C) for subsequent microscopy and metabolic analyses.

### 4.2. Growth Analyses

Stems and leaves were collected and measured to determine stem length (cm), leaf number, and internode length [9].

### 4.3. Sample Preparation to Determine Tissue Structure and Ultrastructure by Microscopy Analysis

#### 4.3.1. Karnovsky’s Fixative Solution

All the microscopic analyses used samples that were fixed in Karnovsky’s solution. This solution contained 2.5% glutaraldehyde and 2% paraformaldehyde in 0.05 M phosphate buffer (pH 7.2). All the reagents used to fix the sample material were of high quality (electron microscopy grade) and purchased from Sigma-Aldrich (Sigma-Aldrich Inc., St. Louis, MO, USA) or EMS (Electron Microscopy Sciences, 1560 Industry Road Hatfield, PA, USA). This preserves all the structures, major molecules, and metabolic components of the organs, tissues, and cells, and allowed further analysis, using different techniques, to be carried out with high precision and accuracy. Regardless of the subsequent methods selected and evaluated in this study, the reliability of this solution was demonstrated.

#### 4.3.2. Embedding in Polyethylene Glycol 6000

Plant stem samples were removed from Karnovsky’s fixative solution, washed three times in distilled water (each time for 5 min), and immersed in 25% PEG 6000 (*w*/*v*) for 24 h at 50 °C in an oven. PEG 6000 was used here. However, it is also possible to use PEG polymers with a molecular mass between 1500 and 6000, to obtain better polymerisation results. Samples were embedded and when the volume of the recipient tissue reached half of the initial volume (after 24–48 h), a solution of 90% PEG was added to the initial volume [3]. After 24–48 h of evaporation, the tissue fragments were embedded in blocks, using 90–100% PEG 6000 (modified by the addition of gum Arabic) [3,24]. The exclusion of gum Arabic resulted in the production of friable blocks, requiring extra care during sectioning using the microtome [3,24]. The blocks were stored at −16 °C before sectioning, to keep them hard and cold. The hardened PEG blocks were bonded to wood blocks using pure or 90% PEG, and transferred to the rotary microtome Leica RM2255 (Leica Inc., Buffalo Grove, IL, USA) for sectioning [3].

#### 4.3.3. Phase Contrast Microscopy Following Embedding in Methyl Methacrylate and Toluidine Blue Staining

Fixed stem segments were subsequently dehydrated in a graded ethanol series (50%, 70%, 80%, 90%, 95%, and 100%) for 1 h at each stage, and embedded in methyl methacrylate Historesin Leica^®^ (Leica Inc., Buffalo Grove, IL, USA) in a 3:1, 1:1, and 1:3 ratio, as well as in pure resin, according to the manufacturer’s instructions. Blocks were stored in polyethylene cases. After 2 days, the blocks were collected and sectioning was performed on a rotary microtome Leica RM2255 (Leica Inc., Buffalo Grove, IL, USA). The 5-µm sections were then dyed with toluidine blue in acetate buffer (pH 4.7). Images were using a Leica ICC50 camera with phase contrast. Anatomical data were quantified from the micrographic images using the Image-Pro Plus^®^ software (Media Cybernetics Inc., Rockville, MD, USA).

#### 4.3.4. Epifluorescence Microscopy Analysis of Stained Cellulose and Lignin

Fixed stem segments were dehydrated in a graded ethanol series (50%, 70%, 80%, 90%, 95%, and 100%) for 1 h at each stage, and embedded in methyl methacrylate Historesin Leica^®^ (Leica Inc., Buffalo Grove, IL, USA). The material was then cut into 5-µm thick sections using a rotary microtome. The sections were double-stained with Calcofluor Bright White 28 (0.01%) and Auramine O (0.01%) [14] for cellulose (blue) and lignin (yellow) staining, respectively. This dual staining method allows cellulosic parenchyma cells to be distinguished from lignified fibres and tracheal elements. Here, the 5-µm sections were immersed in the Auramine O solution (0.01% (*w*/*v*)) for 50 s, washed with distilled water and stained with Calcofluor Bright White 28 (0.01% (*w*/*v*)) for 15 min. Each section was placed between a slide and a coverslip using water and immediately visualised under a Zeiss Axiostar Plus fluorescence microscope (Carl-Zeiss-Promenade, Jena, NA, Germany) with a UV filter attached to an Axiocam digital camera (Carl-Zeiss-Straße, Oberkochen, Germany) [9].

#### 4.3.5. Staining with Phloroglucinol-HCl (Wiesner Reagent)

The Wiesner reagent was prepared using 1% phloroglucinol in hydrochloric acid (18%) moments before use and the sheet was rinsed twice for 5 s. The slides were stained with a few drops of this reagent and tissue samples were analysed using a Leica ICC50 light microscope (Leica Inc., Buffalo Grove, IL, USA). Lignin was stained in different shades of red colour.

#### 4.3.6. Staining with Mäule’s Reagents

Staining using Mäule’s reagents was carried out to detect metabolites and lignin monomers in the stems, as follows [3]. Samples were first treated with 1% KMnO4 aqueous solution for 5 min. Next, they were washed in distilled water and treated with 10% HCl until the sections turned dark brown (5–10 min). The sections were washed again (twice) in distilled water, mounted on slides with concentrated ammonium hydroxide, and immediately photographed. In the tissue samples prepared using this method, syringyl lignin (S-type) stained red, whereas guaiacyl (G-type), and hydroxyphenyl (H-type) lignin stained brown, following with Polyethylene glycol 6000 (PEG 6000) [3].

#### 4.3.7. Identification of Lignin Monomers by Epifluorescence Microscopy Analysis

Stem segments were fixed in Karnovsky’s solution [22] and stored at 4 °C until further processing. The stem samples were then washed in distilled water for 5 min, rehydrated and placed in glass containers containing aqueous polyethylene glycol. Once the PEG 6000 solution reached 25% (*w*/*v*), a 75% (*w*/*v*) PEG 6000 solution was added. When this solution again reached half the total volume, the samples were immersed in a solution of 90% PEG 6000 and gum Arabic [3]. The stem fragments were placed in cassettes mounted on a wood base with adhesive tape and immediately frozen at −16 °C. Next, the samples were removed from the freezer, cut using a manual rotary microtome Leica RM2235 (Leica Inc., Buffalo Grove, IL, USA) (thickness 25–35 µm) and floated on warm water (between 35 and 50 °C). Staining was performed using 1% (*w*/*v*) astra blue and safranin/basic fuchsin, and each section was then mounted between a slide and a coverslip containing 50% glycerine or water. Digital images were obtained from an EKB-2F epifluorescence microscope (Eikonal Inc., São Paulo, SP, Brazil) or a Zeiss Axiostar Plus fluorescence microscope (Carl-Zeiss-Promenade, Jena, NA, Germany) with a UV filter attached to a digital camera Axiocam (Carl-Zeiss-Straße, Oberkochen, Germany), both set at the violet excitation wavelength (400 nm). Captured images were processed using the Bel Eurisko (Bel Photonics Inc., Monza, Italy) or Zeiss software. Using the Image-Pro-Plus^®^ v.4.5 software, qualitative and quantitative analyses were carried out to determine changes in vascular cylinder morphology. All analyses were performed using cross-sections of the stem and focused on the vascular cylinder, which is composed of tracheary elements (tracheids and vessel elements), fibres (fibre-tracheids and libriform fibres), and parenchyma cells. In addition, a few cells of the primary xylem (e.g., protoxylem) were observed on the slides. The term fibre-like cells refer to all the types of fibre cells present on the axial system, except highly differentiated vessel elements analysis [38].

#### 4.3.8. Scanning Electron Microscopy

Samples analysed by scanning electron microscopy (SEM) were fixed with Karnovsky solutions and subsequently embedded with a cryoprotectant (glycerol) at different concentrations (10%, 20%, and 30%) until they sank. The fragments were then immersed in liquid nitrogen and fractured using a scalpel blade. The fragments were then placed in a container with distilled water and dehydrated using a graded series of acetone (30%, 50%, 70%, and 90%) for 1 h at each concentration, and finally immersed in 100% acetone three times, each for 10 min. Samples were dried on a CPD-030 Bal-Tec critical point dryer (Bal-Tec AG, Balzers, Liechtenstein, Germany), assembled into stubs and metallised with gold on a MED010 Balzers evaporator (Bal-Tec AG, Balzers, Liechtenstein, Germany). Samples were observed under a Quanta 250 scanning electron microscope at 25 kV (FEI Company, Hillsboro, OR, USA) to detect xylem fibre-like-cell and vessel elements.

#### 4.3.9. Transmission Electron Microscopy

For transmission electron microscopy (TEM) analysis, fixed stems samples were post-fixed for 1 h using 1% osmium tetroxide in 0.05 M cacodylate buffer. The samples were then contrasted in bloc with 0.5% uranyl acetate overnight, dehydrated in a graded acetone concentration series (30%, 50%, 70%, 80%, 90%, and 100% (three times)), infiltrated and then polymerised into Spurr low viscosity epoxy resin. Sections (70-nm thick using a diamond knife) were obtained using an ultramicrotome (MTX Powertome X, Boeckeler Instruments, RMC Products, Phoenix, AZ USA) and contrasted with 3% uranyl acetate and lead citrate in grids with 200–300 mesh size. Analyses were performed using a transmission electron microscope (JEOL JEM-1400; Leica Microsystems Inc., Buffalo Grove, IL, USA) [26] equipped with a digital camera set at 100 kV.

Quantification of images of the region of the vascular cylinder were obtained in 10 different stems (from 10 different plants) cut. Subsequently, 10 different images were analysed, and 10 regions obtained randomly from the cell wall of the vascular xylem were measured from each image. This practice is commonly used with the help of software such as ImageJ or Image-Pro-Plus^®^.

### 4.4. Preparation of Protein-Free Cell Walls (PFCWs) for Simple Metabolic Analysis

A total of 150 mg of stem powder was weighed into 2-mL microtubes. At each step, samples were washed five times with 50 mM of potassium phosphate buffer (pH 7.0), five times with Triton X-100 (pH 7.0), four times with 1 M NaCl (pH 7.0), four times with distilled water and three times with acetone. Centrifugation was performed at 15,000 rpm for 3 min after each step. Finally, the pellets were oven-dried at 60 °C for 24 h. The resulting material was considered as the PFCW fraction [29]. PFCWs are free of water-soluble compounds (e.g., polar and apolar compounds) [9].

### 4.5. Determination of Lignin Content in PFCW

Lignin content was measured in 20 mg of PFCW using the acetyl bromide method [29]. In a new tube, 0.13 mL of a freshly prepared acetyl bromide solution (25% (*v*/*v*) acetyl bromide/glacial acetic acid) was added and incubated at 70 °C for 30 min. After total digestion, the samples were quickly cooled on ice and mixed with 0.24 mL of 2 M NaOH, 0.02 mL of 5 M hydroxylamine-HCl and 1.6 mL of glacial acetic acid, to achieve complete solubilisation of the lignin extracts. The sample was centrifuged at 1400× *g* for 5 min. Quantification of the supernatant was determined against a standard curve generated for alkaline lignin and the absorbance value (ε) was 22.1 g L^−1^ cm^−1^ at λ280 nm. Microplate reading was performed using a Biochrom Asys UVM-340 Microplate-Reader of 96-wells with ScanPlus VisibleWell^®^ Software (Biochrome Ltd., Milton Road, Cambridge, England). The results were expressed as mg lignin g^−1^ PFCW.

### 4.6. Quantification of Cellulose

Cellulose quantification was performed as described in [28,39]. Stem samples were incubated at 70 °C for 1 h. Ethanol was replaced with acetic/nitric acid for extraction. This solution was then discarded, and the samples were washed with distilled water. Next, the water was replaced with a freshly prepared anthrone-sulphuric acid solution. The samples were analysed using a microplate reader Biochrom Asys UVM-340 (Biochrome Ltd., Milton Road, Cambridge, England) at λ620 nm. Cellulose concentration was expressed as its equivalent to glucose concentration (µmol glucose g^−1^ DW) against a standard curve.

### 4.7. Total Soluble Phenolic Compounds

Total soluble phenol (PhC) quantification was carried out with modifications [40]. Each sample was crushed in 2 mL of cold methanol and centrifuged at 15,000 rpm for 15 min, and the methanolic extract was transferred to a new 1.5 mL tube. Phenolic quantification was initiated by the addition of 150 μL of methanolic extract, 70 μL of Folin-Ciocalteu reagent (1 M), 140 μL of Na_2_CO_3_ (3.56 M), and 850 μL of deionised water. The samples were incubated in the dark for 50 min and were then centrifuged for 120 s at 15,000 rpm, followed by analysis on a microplate reader Biochrom Asys UVM-340 (Biochrome Ltd., Milton Road, Cambridge, England) at λ725 nm. The data were generated from UV-Star^®^ 96-well microplates containing 200 µL of prepared extract per well, using the Asys UVM-340 microplate reader linked to the ScanPlus VisibleWell^®^ software (Biochrome Ltd., Milton Road, Cambridge, England). The equivalent PhC concentration was determined using gallic acid as a reference (Y = 87.651x + 1.6515; R^2^ = 0.993).

### 4.8. Enzymatic Saccharification to Release Reducing Sugars

Soluble sugars were removed from the dry biomass using 50 mg of biomass in centrifuge tubes with 2 mL of 80% alcohol per wash. The tubes were incubated at 55 °C for 4 h. Before proceeding with the experiment, the sugar content of the insoluble precipitate was determined by the phenol-sulphuric acid method. The sample was placed in the oven at 60 °C and 15 mg of the insoluble precipitate was weighed and incubated with 81 µL of crude xylanase extract (Megazyme Ltd., Bray, Wicklow, Ireland) and 919 µL of 50 mM acetate buffer (pH 5.0) for 4 h at 50 °C [41]. The reaction was stopped using 100 µL of 1% DNS (dinitrosalicylic acid) and immediately boiled at 100 °C for 5 min. The mixture was then placed on ice, and 800 µL of distilled water was added. All readings and quantifications were generated from 96-well UV-Star^®^ microplates containing 200 µL of prepared extract per well, using the Biochrom Asys UVM-340 (Biochrome Ltd., Milton Road, Cambridge, England).

### 4.9. Statistical and Graphical Analyses

The generated data were subjected to variance homogeneity analysis using Barlett’s test, for all variables. The quantitative data obtained were subjected to the Student’s *t*-test, with values considered significant at an error probability of less than 5%. All univariate statistical analyses and graphics were prepared using the Microsoft Excel^®^ (Microsoft Office Professional 2019, Mountain View, CA, USA) and CorelDraw 2020^®^ (Corel Corp., Ottawa, ON, Canadian) software packages. In addition, correlation analysis was performed and correlograms were generated using the R-software Corrplot package (R-Core Team, 2021). A schematic representation of the data was generated using Bio-Render^®^ 2021. The data obtained from each sampling unit (plant) were subjected to multivariate analysis through principal component analysis (PCA) using the Statistica^®^ 10.0 software (Statsoft Inc., Tulsa, OK, USA).

## 5. Conclusions

In the present study, far-red light supplementation induced a series of changes in tobacco plants. Changes at the structural, ultrastructural, and biochemical levels were quickly analysed using different microscopic techniques and the composition of the primary and secondary cell wall was determined. In this plant methods communication, we describe the main techniques used, i.e., fluorescence, light microscopy, as well as scanning and transmission electron microscopy, in addition to the methods employed for measuring the levels of lignin, cellulose, phenolic compounds and total reducing sugars. Collectively, these techniques can be used to understand the dynamics of perception and photodevelopment in plants, in response to different environmental conditions. We also present a flowchart for the implementation of routine analyses in laboratories that wish to employ these anatomical and molecular tools. Our data suggest that researchers, especially those investigating the cell wall, use these different microscopy tools to better understand changes at the organ, tissue, and cellular levels. These techniques are mostly relatively simple, quick, and inexpensive to perform, and can provide valuable information on plant cell wall development. In addition, the application of microscopic techniques benefits the future of plant research by directing resources to the more sophisticated analytical “omic” technologies, such as metabolomics, glycomics, proteomics, and genomics for the study of cell walls.
